# Behavioral and Electrophysiological Responses of the Fringed Larder Beetle *Dermestes frischii* to the Smell of a Cadaver at Different Decomposition Stages

**DOI:** 10.3390/insects11040238

**Published:** 2020-04-10

**Authors:** Clément Martin, Damien Minchilli, Frédéric Francis, François Verheggen

**Affiliations:** Gembloux Agro-Bio Tech, TERRA, University of Liège, Passage des Déportés 2, 5030 Gembloux, Belgium; cmartin@uliege.be (C.M.); minchdam@gmail.com (D.M.); frederic.francis@uliege.be (F.F.)

**Keywords:** Dermestidae, electrophysiology, forensic entomology, necrophagous coleopteran

## Abstract

A cadaver is colonized by a wide diversity of necrophagous insects. It is well documented that Dipterans are attracted by the volatile organic compounds (VOCs) released by a corpse during the first minutes following death. Coleopterans are known to be attracted by highly decomposed cadavers, but have received less attention regarding the olfaction-based mechanisms underlying these interactions. In the present study, we impregnated gauzes with VOCs collected from each decomposition stage of dead rats: fresh, bloated, active, and advanced decay. We collected the VOCs released by the gauze and confirmed what was previously know from the literature: the decomposition stages are associated with contrasting chemical profiles. We exposed *Dermestes frischii* Kugelann (Coleoptera: Dermestidae) male and female antennae to the same gauzes and found that stronger electrical responses were recorded when using the smell of the advanced decay stage. Finally, we performed two choices behavioral assays. Females showed no preference for the four decomposition stages, while males were attracted by the smell associated with active and advanced decay stages. These results suggest that specific VOCs released by a decaying body guide necrophagous coleopterans to their feeding site. Whether *D. frischii* males release pheromones to attract females remains to be tested.

## 1. Introduction

The decomposition of a corpse is associated with the release of hundreds of chemicals including volatile organic compounds (VOCs) [1,2,3,4,5]. The abundance and composition of the cadaveric volatilome are impacted by biotic (e.g., microorganism, insects) and abiotic factors (e.g., temperature, humidity), but also differs according to the decomposition stage the cadaver is undergoing (fresh, bloated, active decay, advanced decay, and dry remains) [6,7,8,9,10,11,12]. For instance, a cadaver under the bloated stage typically releases higher amounts of alcohols, ketones, amines, and carboxylic acids, while a corpse under active decay releases higher quantities of aromatic compounds (e.g., indole) [10,11,12,13,14].

Various necrophagous insect species use cadaveric volatile compounds to find a feeding and/or mating site [15]. They are attracted to the cadaver in a relatively predictable sequence called the entomofaunal succession [16]. Dipterans are the first colonizers, with blowflies (Calliphoridae) arriving on the corpse within the first minutes following death. They are usually followed by flesh flies (Sarcophagidae) and houseflies (Muscidae). The importance of putrefactive sulfur-based compounds (e.g., dimethyl trisulfide, dimethyl disulfide) in the attraction of blowflies was confirmed in various studies [17,18,19,20]. Dipterans are not alone on the corpse. They are quickly joined by coleopterans. However, coleopterans are more likely to arrive on a corpse during the later stages of decomposition: active decay, advanced decay, and dry remains [16,21,22,23]. Through these stages, the corpse dries more and more. As a consequence, dry protein-rich organic matter (e.g., skin and tissues) are most abundant and are known to be the favorite feeding source for coleopterans, such as Dermestidae [23,24,25]. The number of chemo-ecological studies investigating coleopteran species is relatively low compared with those on blowflies, and among them, Silphidae are the most studied [15]. Silphidae (e.g., *Thanatophilus sinuatus*) have sensitive chemosensors located on their antennae adapted to detect cadaveric organic compounds [26,27], and both males and females respond behaviorally to polysulfide compounds, such as dimethyl disulfide [28,29]. Dermestidae have received very limited attention. However, it has been documented that *Dermestes maculatus* use saponificated triacyl glycerides and long-chained fatty acids to locate a cadaver, including benzylbutyrate [15,30,31]. Males *D. maculatus* arrive on a corpse before females [30]. Females are then attracted by a combination of cadaveric volatiles and possibly the odor emitted by males [30]. However, Dermestidae can also be found on an early decay cadaver, suggesting their ability to detect cadaveric VOCs associated with early stages [23,32,33].

In the present research, we aimed at evaluating the ability of *Dermestes frischii* (Coleoptera: Dermestidae) to perceive and forage for a cadaver using the odor associated with all stages of decomposition. To the author best knowledge, this is the first study to investigate the olfaction-based mechanisms underlying the interactions between *D. frischii* and a cadaver.

## 2. Materials and Methods

### 2.1. Insect Rearing

*D. frischii* were mass-reared in a sealed plastic box (50 × 30 × 40 cm) containing wood chips and polystyrene. The box was permanently placed in an incubator, in the dark, at 30.0 ± 0.1 °C and at controlled humidity (40 ± 1%) [12]. They were fed twice a month with a dead rat which was used as oviposition site and food source for the larvae [12]. *Lucilia sericata* Meigen (Diptera: Calliphoridae) were purchased as larvae in a fishery (Lacroix Pêche, Perwez, Belgium). They were placed in a group of 100 individuals in a Petri dish (d = 20 cm) at 23 ± 1 °C under a 8:16 h light:dark photoperiod. They were daily fed with pig liver until pupation [34]. Pupae were collected and placed in a net cage (30 × 30 × 30 cm) until emergence. Adults were sexed and placed separately in similar net cages.

### 2.2. Rat Decomposition

Four male laboratory rats (408.05 ± 30.15 g) (*Rattus norvegicus,* Berkenhout, 1769) were raised and euthanized at the Faculty of Veterinary Medicine of the University of Liège (ethic agreement n°18-2021), by CO_2_ asphyxia, before being frozen until the start of the experiment. Each rat was defrosted in a hot water bath (≈ 40 °C) and let to decompose inside 30-L glass cylindrical tanks. In order to mimic natural decomposition, five couples of newly emerged *L. sericata* were introduced into all-glass tanks [12]. Water and sugar were also added in each tank to ensure the survival of adult flies. A fifth tank was also set up as a control, under the same conditions but containing no rat. Temperature and relative humidity were measured using Data logger^®^ sensors (Lutron^®^, Taipei, Taiwan).

### 2.3. Odor Collection and Analysis

In order to collect the VOCs released during the different decomposition stages, sterile gauzes (n = 90 per tank) (1/3, 5 × 5 cm; Stella^®^; Lohmann & Rausher s.a.; Liège-Rocourt; Belgium) were suspended using a fishing line in the headspace of each tank, as suggested in previous reports [35,36]. The decomposition stage (i.e., fresh, bloated, active and advanced decay) of each rat was evaluated daily [10]. At the start of each decomposition stage, new gauzes were placed for 24 h at a distance of 10 cm from the cadaver, before being removed and stored in sealed glass Petri dishes (d = 20 cm) placed at −20 °C.

In order to confirm that the four groups of sterile gauzes are carrying contrasted blends of cadaveric VOCs, half a gauze was directly inserted in an empty VOC sampling tube (length 8.9 cm; external diameter 0.64 cm; Markes international^®^; Llantrisant; UK) for gas chromatography analyses (Shimadzu; Kyoto; Japan). VOCs were thermodesorbed at 280 °C during eight minutes, before being cryofocused by Peltier effect at −20 °C, and heated at 280 °C to be injected on a capillary column (5% diphenyl; 30 m × 0.25 mm I.D.; film thickness 0.25 µmm) (Filter service^®^; Eupen; Belgium). The gas chromatograph setup, temperature program, quantification and identification methodology were previously described in Martin et al., 2019 [12].

### 2.4. Electroantennography

Electroantennography assays were performed to compare the ability of *D. frischii* to discriminate the VOC blend from each decomposition stage, by measuring the total antennal response to each blend. The setup used was completely described in Verheggen et al. (2008) [37]. Glass electrodes were shaped to fit Coleoptera antennae [29,38,39]. The head of each insect was cut off from the entire body and connected to the ground electrode, while the extremities of both antennae were placed in contact with the working electrode. The electrical responses of ten males and ten females were recorded. Six stimulations were performed in random order on each antenna, separated by 30 s: Each antenna was exposed to the VOCs of the four decomposition stages (provided by a 0.25 × 0.5 cm piece of sterile gauze) as well as to a positive and negative control. The positive control consisted of 110 ng of dimethyl disulfide (purity > 99%; Sigma-Aldrich^®^; Saint-Louis, MO, USA) placed on a piece of sterile gauze. The negative control was a piece of gauze taken from the control tank.

### 2.5. Behavioral Assays

**Figure 1:**
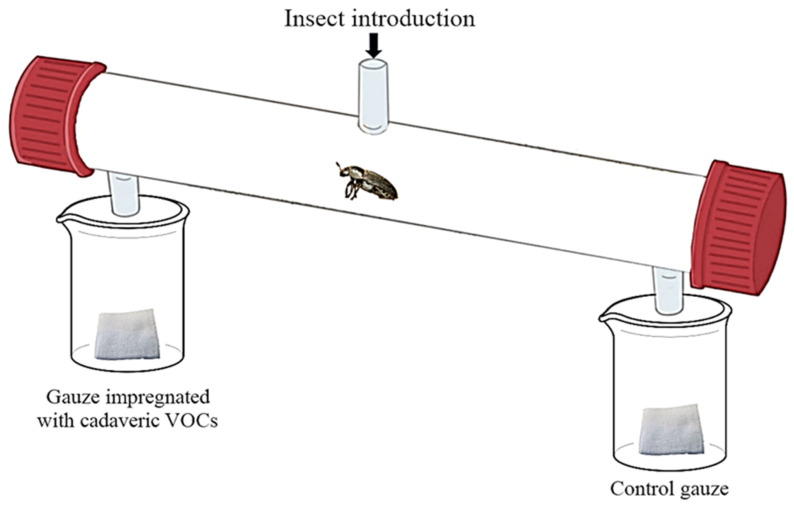
The dual choice olfactometer used to assess the preferences of *Dermestes frischii* for the volatile cues associated with each decomposition stage.

The bioassays were performed using a glass cylindrical olfactometer (32.0 cm long; 3.6 cm internal diameter) (Figure 1) [40,41]. The central opening (GL14) of the olfactometer allowed a single insect to be introduced. One additional opening was located at both ends of the olfactometer, connected with beakers containing a piece of gauze (0.25 cm^2^). Each piece of gauze was used only once. Parafilm covered both beakers to concentrate the odor into the olfactometer. The odor of each decomposition stage was tested against a control (clean gauze). The positions of the control and the odor-impregnated gauzes were randomly assigned. Before being introduced in the olfactometer, each insect (n = 90 males and 90 females for each decomposition stage) was placed on the ice for 30 s, to reduce escape and stress effects. The insect was allowed to move for 30 min in the olfactometer. Ten olfactometers were used at the same time. All behavioral tests were performed in the dark to avoid light bias. Each individual was tested once. Between each bioassay, the olfactometers were cleaned with pentane (purity = 99.8%; Sigma-Aldrich^®^; Saint-Louis, MO, USA).

### 2.6. Statistical Analysis

All the results were statistically processed using RStudio^®^ software (3.6.1 version). Behavioral data were analyzed with a generalized linear mixed model (GLMM) (function “glmer”, R-package “lmertest”) [42] to evaluate the effect from two random factors: sets of trials and rats. Since these effects were not significant, a simple generalized linear model (GLM) was used (function “glm”, R-package “lme4”) to assess the impact of sex and stage of decomposition. In order to highlight differences between the odor profiles of the different stages of decomposition, a principal component analysis (PCA) followed by a multivariate analysis by permutation test (PERMANOVA) were performed.

## 3. Results

**Figure 2:**
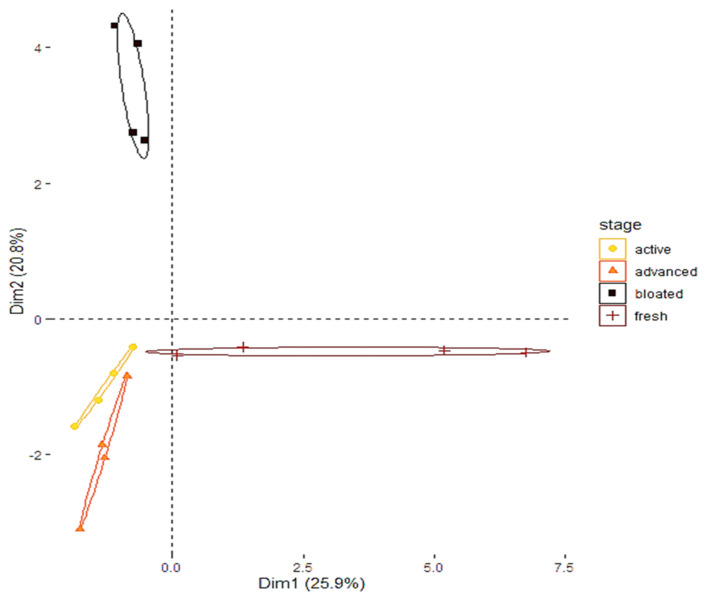
Principal component analysis displaying the contrasted composition of the cadaveric COVs collected from each stage of decomposition.

The PERMANOVA analysis shows that the volatile compositions of the sterile gauzes associated with each decomposition stage were statistically different (F_3,12_ = 3.648; *p* = 0.001). This conclusion is displayed by the PCA (Figure 2). Because of the specific method used to trap the volatile compounds (i.e., impregnation of sterile gauze), many VOCs were identified under the limit of quantification. Carboxylic acids (e.g., propionic acid, butanoic acid, acetic acid) and amines (e.g., Pyrazine, tetramethyl) were specifically identified during the advanced decay stage. Indole was only detected during the active stage. The bloated stage was characterized by alcohol (e.g., ethanol) and alkanes (e.g., eicosane). Alkanes were also detected during the fresh stage.

**Figure 3:**
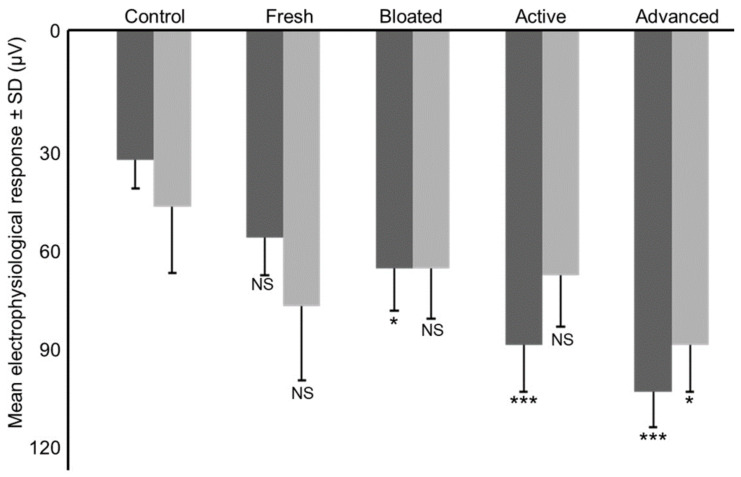
Mean electrophysiological response of male (dark grey) and female (light grey) antennae towards the scent of the different stages of decomposition (μV ± SD). *** *p < 0.001*; ** *p < 0.01*; * *p < 0.05*; NS *Not Significant*.

Male antennae produced different electrical responses to each of the four stages of decomposition (F_4,36_ = 8.262; *p* < 0.001). With the exception of the fresh stage, the smell of all decomposition stages elicited electrical responses different from the control (fresh: t_36_ = −1.725; *p* = 0.093; bloated: t_36_ = −2.415; *p* = 0.021; active decay: t_36_ = −4.140; *p* < 0.001 and advanced decay: t_36_ = −5.175; *p* < 0.001) (Figure 3). Female antennae did elicit response only for the advanced stage of decomposition (t_36_ = −2.618; *p* = 0.013) but not for the other stages (fresh: t_36_ = −1.891; *p* = 0.067; bloated: t_36_ = −1.164; *p* = 0.252; active decay: t_36_ = −1.309; *p* = 0.199) (Figure 3).

**Figure 4:**
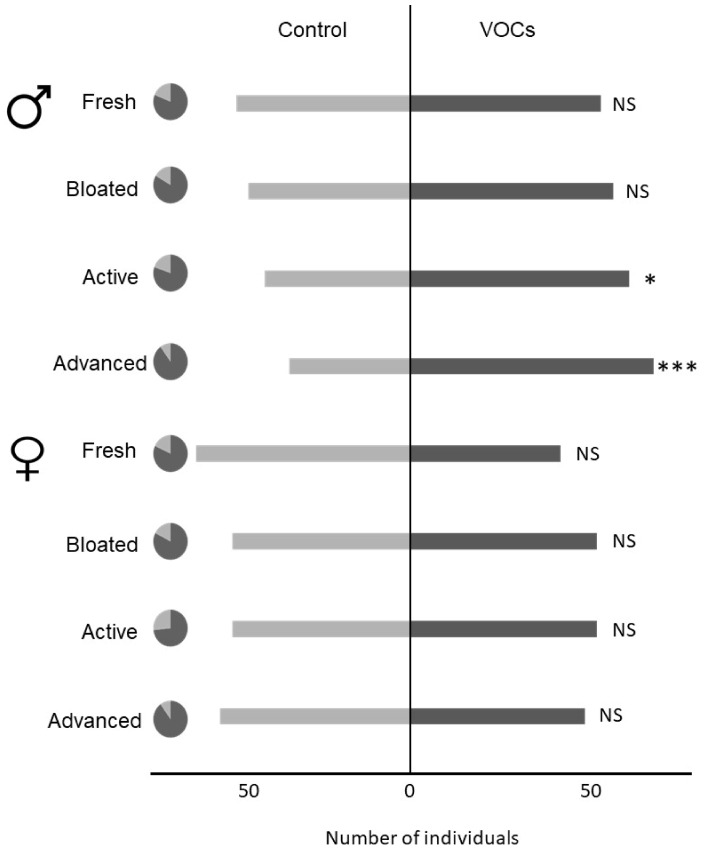
Female and male behavioral responses to the volatile cues associated with each decomposition stage. Dark grey areas in pie charts display proportions of responding individuals. *** *p < 0.001*; ** *p < 0.01*; * *p < 0.05*; NS *Not Significant*.

None of the random factors (i.e., rat used for VOCs collection and batch of bioassays) impacted beetles’ behavioral responses. Since males and females did not exhibit the same behavioral responses (F_1,715_ = 8.562; *p* = 0.003), they were analyzed separately (Figure 4). Males responded to the VOCs associated with a cadaver under active (*p* = 0.050) and advanced decay (*p* = 0.001) decomposition, but did not respond to earlier decomposition stages including fresh (*p* = 0.673) and bloated (*p* = 0.293). Females were not attracted to the volatile cues associated with any decomposition stage (fresh: *p* = 0.093; bloated: *p* = 0.883; active decay: *p* = 0.883; advanced: *p* = 0.673).

## 4. Discussion

The main objective of the present study was to evaluate the ability of *D. frischii* to perceive and respond to the volatile compounds associated with the different stages of decomposition of a cadaver. It aimed at confirming a common observation: necrophagous beetles are more likely to colonize a corpse during the advanced decay stage [43,44,45,46,47,48].

The electrophysiological experiment revealed differences in the ability of the olfactory apparatus of females and males to perceive the chemical cues released during the entire decomposition process. Unfortunately, really poor information is available on the structural characterization of the diversity of sensilla in this species. Such data may explain why females perceive the chemical compounds released during the advanced decay stage, while males perceive the smell of a cadaver at the earliest stages of decomposition. Males and females in the species *Thanatophilus sinuatus* Fabricius, a necrophagous beetle belonging to the Silphidae family, were already shown to have different abilities to perceive cadaveric compounds [29]. However, here also, the structural characterization of the antennae was not performed.

We wanted to have sterile compresses impregnated with cadaveric odors to be able to carry out electrophysiological and behavioral assays. This method of volatile capture is, however, not adapted to perform a fine screening and quantification of the volatile organic compounds released by dead rats. As a consequence, many VOCs were identified but were found to be under the limit of quantification. Based on the quantifiable compounds, all decomposition stages were shown to have distinct volatile signature, confirming many previous reports [12,21].

The behavioral trials showed that even if males can detect compounds from the early stages of decomposition, they are only attracted by a cadaver under active and advanced decay stages. On the other hand, even if females perceive cadaveric VOCs from the advanced stage, they are not attracted by this stage. These information confirm the common observation that necrophagous coleopterans prefer colonizing advanced decay corpses [43,44,45,46,47,48].

Our behavioral and electrophysiological data confirm previous field observations stating that males are the first to colonize a corpse. One could raise the hypothesis that females do not respond to the smell of a cadaver unless they detect the presence of males. Upon encounter with a cadaver, males might be releasing pheromones that attract females and possibly reinforcing males’ attraction. This pheromonal cue could act in synergy with cadaver VOCs, to inform females about the presence of a mating site where they can both reproduce or lay their eggs. Such a chemical communication has been shown in *Nicrophorus vespilloides* Herbst (Coleoptera: Silphidae) and *D. maculatus* [27,30,49]. The existence and composition of such a pheromone is yet to be characterized [27,50]. Futures studies using two-dimensional gas chromatography could help to detect the pheromonal compounds in such a complex blend of volatile molecules [51].

While *D. frischii* males perceive the volatiles from a cadaver at any decomposition stage, they do not behaviorally respond to each of them. How *D. frischii* differentiates between the early stages and the latter stages remains to be solved. Some specific chemical compounds might be used to evaluate the decomposition stage of the cadaver. While some compounds are continuously released during the entire decomposition process, some specific compounds are associated with a specific decomposition stage [10,15]. Sulfured compounds (such as dimethyl trisulfide and dimethyl disulfide) are released during the entire process of decomposition and are probably responsible for the electrical depolarization recorded from male and female antennae [10,12,52,53]. Indeed, several other necrophagous coleopterans are able to perceive these compounds. For instance, Silphidae commonly use these compounds to target a potential corpse to colonize [15,27,28,54]. However, since *D. frischii* is attracted by specific decomposition stages and not all of them. This might be explained by the perception of additional compounds and/or by the ability of *D. frischii* to perceive variation in the relative concentrations of each volatile compound. Some chemicals may be attractive or repellent to necrophagous insects, according to their concentration [15,29]. Also, some necrophagous coleopterans respond to saponificated long chain fatty acids such as benzylbutyrate (e.g., *D. maculatus*) [30]. These compounds are released during the later stages of decomposition and could act in synergy to inform *D. frischii* to the best moment to colonize the corpse [10,15].

## 5. Conclusions

*D. frischii* male and female antennae elicited strong electrical depolarization during exposure to the smell of a cadaver at the advanced decay stage. While female *D. frischii* were not attracted to the smell of a cadaver (in any stage of decomposition), males were attracted by the smell associated with the active and advanced decay stages. These results suggest that specific VOCs released by a decaying body guide necrophagous coleopterans to their feeding site. These results lend support to the hypothesis that females do not respond to the smell of a cadaver unless they detect the presence of males, releasing either sex or aggregation pheromones.
